# Fatigue Behavior of Woven Glass Fiber-Reinforced Epoxy Laminated Insulation (IEC 60893 EPGC 203) for High-Voltage Applications

**DOI:** 10.3390/polym18141690

**Published:** 2026-07-09

**Authors:** Oguzkan Senturk, Rupesh Daripa, Vivekkumar Chaubey, Tirdad Boroomand, Tobias Stirl, Rajeev Gupta

**Affiliations:** 1 GE Vernova Grid Solutions, Stafford ST16 1WT, UK; 2 GE Vernova Grid Solutions, Vadodara 390013, India; 3 GE Vernova Grid Solutions, 41065 Mönchengladbach, Germany

**Keywords:** epoxy, fatigue, glass fiber, high voltage, mechanical testing

## Abstract

This study investigates the mechanical performance and fatigue behavior of a woven glass fiber-reinforced epoxy laminated composite classified as IEC 60893 EPGC 203, widely used in structural insulating components for high-voltage (HV) equipment. With evolving energy infrastructures introducing dynamic and cyclic loading conditions, understanding the long-term mechanical reliability of such materials has become increasingly important. A comprehensive experimental program was conducted, including flexural, compressive, tensile, Charpy impact, and fatigue tests. Mechanical properties were evaluated according to relevant ISO standards at room temperature and 120 °C to assess temperature-dependent performance. Fatigue tests were performed under fully reversed loading conditions (R=−1), and stress–life (S-N) curves were established. The results revealed notable reductions in strength and stiffness at elevated temperature, together with progressive damage accumulation under cyclic loading. Failure features observed after fatigue testing were correlated with static mechanical properties to improve understanding of degradation behavior. The study is limited to the mechanical and fatigue characterization of EPGC 203 and does not include dielectric evaluation. Therefore, its relevance to HV applications is considered from the standpoint of the mechanical reliability of structural insulating components. The findings provide essential insights into the durability and reliability of EPGC 203 composites under realistic service conditions.

## 1. Introduction

Addressing the climate crisis has become an urgent global priority, driving the rapid expansion of clean and renewable energy systems that are essential for ensuring reliable, affordable, and sustainable power, as well as improving overall quality of life. Among these, offshore energy is one of the fastest-growing clean and renewable energy solutions [1]. While current offshore projects are predominantly based on fixed-bottom installations in shallow waters, more than 80% of the global wind resource potential lies in deeper regions where such solutions are not economically viable [2]. This has led to the development of floating offshore technologies, where substations and associated infrastructure are installed on floating platforms. Floating offshore solutions can capture more wind energy but harvesting more energy comes with its own challenges, such as shocks and constant vibrations from wind and waves [3,4]. These conditions introduce additional mechanical stresses and potential failure mechanisms, particularly fatigue-related failure, which significantly affects the structural reliability and long-term performance of offshore energy systems [5,6,7].

In this context, HV and HVDC transmission systems are essential for power networks, particularly for long-distance energy transfer and renewable energy integration [8,9]. Key components such as transformers, bushings, and circuit breakers of electrical infrastructure ensure the safe transmission and distribution of electrical energy across transmission lines, substations, and power equipment [10]. These key components rely on insulation systems that must provide electrical insulation, thermal stability, and mechanical strength with increasing operational demands and service conditions [11,12]. While conventional HV equipment has traditionally been designed under static or quasi-static loading assumptions, insulation materials are required to withstand long-term exposure to electrical stress, elevated temperatures, and environmental factors, which directly influence the reliability and lifetime of the equipment [13,14,15,16,17,18,19]. In addition to emerging applications such as offshore installations, modular converter stations and transport-induced constraints also introduce significant cyclic and dynamic mechanical loads [20].

Polymer-based composite materials, particularly glass/epoxy laminates and silicone rubber composites, are widely used in HV applications due to their advantageous combination of dielectric strength, weight, and mechanical performance. However, long-term exposure to electrical, thermal, and environmental stresses such as humidity, ultraviolet radiation, and pollution can lead to degradation mechanisms including embrittlement, cracking and erosion [21]. Experimental studies on polymer-based composite insulation materials in HV systems have predominantly focused on multi-stress aging conditions and their impact on mechanical, thermal, and dielectric performance [22,23]. Despite the increasing amount of research on HV insulation systems, the fatigue behavior of structural insulation materials is still not well known. This is particularly important for load-bearing composite components used in HV transmission structures, where cyclic mechanical stresses can significantly affect long-term performance [24].

The fatigue strength of fiber-reinforced polymer composites, particularly glass fiber-reinforced epoxy composites, has been extensively studied and is characterized by progressive damage accumulation mechanisms such as matrix cracking, fiber–matrix debonding, and interlaminar delamination [25,26,27,28]. Environmental factors such as temperature and moisture further accelerate fatigue degradation and reduce service life [29], while complex loading conditions, including spectrum and multiaxial loading, significantly influence damage evolution and fatigue life [30,31]. In addition, interfacial properties and material architecture play a critical role in determining fatigue strength and crack propagation behavior [32,33]. From a modeling and experimental perspective, the complex and anisotropic nature of composites makes fatigue prediction challenging, often requiring extensive experimental characterization and conservative design approaches [34]. Interlaminar fatigue behavior has also been identified as a critical failure mode, particularly in laminated composites, where delamination significantly contributes to structural degradation [35]. In addition, hybrid composite systems have shown that interfacial properties and fiber architecture play a crucial role in fatigue resistance and damage evolution [36]. Recent studies further highlight the importance of multiaxial and non-proportional fatigue loading, which more accurately represents real service conditions and leads to complex damage interactions and accelerated degradation [37]. Fatigue damage models have been developed to capture stiffness degradation and predict fatigue life, emphasizing the relationship between loading level, damage evolution, and residual mechanical properties [38]. Additionally, the role of fiber–matrix interphase has been shown to significantly affect fatigue life, with improved interfacial bonding leading to enhanced resistance to crack propagation [39].

In HV and HVDC equipment, some insulation materials are required to fulfill a dual function by acting both as electrical insulation and as structural support [40]. In components such as insulating spacers, transformer support members, structural parts of bushings, switchgear elements, and converter-related assemblies, mechanical degradation under cyclic loading may indirectly affect electrical reliability by causing cracking, delamination, stiffness reduction, or dimensional instability [41]. Such changes may alter load-carrying capability, electrical clearances, and local electric field conditions in service [42]. From this perspective, the fatigue resistance of structural insulation-grade laminates is directly relevant to component design and lifetime assessment, especially in applications exposed to vibration, transport loads, thermal cycling, and offshore-induced dynamic loading.

Despite these extensive studies, most existing research has focused on general-purpose structural composites rather than industrial insulation-grade laminates specifically used in electrical equipment. Comparatively limited attention has been given to epoxy glass laminates such as IEC 60893 EPGC 203, which are widely used as structural insulating materials in HV and HVDC applications. As a result, the available knowledge regarding their fatigue performance under conditions relevant to service loading remains limited. This represents an important gap, since reliable mechanical data are needed to support the structural design, material selection, and lifetime assessment of insulating components subjected to repeated loading.

Therefore, the present study aims to provide a comprehensive experimental characterization of the mechanical and fatigue behavior of EPGC 203 epoxy glass laminated composites. A systematic test program including tensile, flexural, compressive, impact, and fatigue loading was carried out under room temperature and elevated temperature conditions. Particular emphasis was placed on stress–life (S-N) behavior, temperature-dependent performance, and damage evolution mechanisms. The results are expected to contribute to a better understanding of fatigue-driven degradation in EPGC 203 structural insulating laminates and to support improved mechanical design methodologies and lifetime assessment of HV and HVDC components operating under realistic service conditions. However, the present study is limited to the mechanical and fatigue characterization of the investigated material and does not include experimental dielectric evaluation. Therefore, the relevance to HV/HVDC applications is considered here from the standpoint of mechanical reliability of structural insulating components, while final engineering qualification should also include electrical and thermal criteria such as dielectric strength, insulation resistance, tracking resistance, and thermal class, as required by the intended application and applicable standards. The findings are not intended to replace electrical qualification data, but rather to complement them in applications where mechanical durability is a critical design requirement.

## 2. Materials and Methods

### 2.1. Materials

The material investigated in this study was a woven glass fiber-reinforced epoxy laminate designated as EPGC 203 according to IEC 60893-1 [43]. The reinforcement architecture consisted of a 0°/90° glass fabric embedded in an epoxy resin matrix. According to the material supplier, the laminate contained approximately 70 wt.% glass fiber reinforcement and exhibited a density of 1.85 g/cm^3^. The reported T_g_ was 160 °C, as determined according to ISO 11359-2 [44]. Test specimens were machined from 6 mm thick laminated plates using a 3D CNC router to ensure dimensional accuracy and repeatability. Material specifications are summarized in Table 1. Detailed information regarding the curing/post-curing history and void content was not available to the authors due to supplier confidentiality and therefore could not be reported in the present study.

### 2.2. Experimental Methods

Mechanical and fatigue experiments of the EPGC 203 were performed in a temperature (25 ± 2 °C)- and humidity (50 ± 5%)-controlled laboratory through a comprehensive experimental program. All tests were conducted in accordance with relevant international standards to ensure the reliability and comparability of the results. All specimens were conditioned for 24 h in accordance with IEC 60212 [45] (temperature 23 ± 2 °C and relative humidity 50 ± 5%), prior to testing.

**Figure 1:**
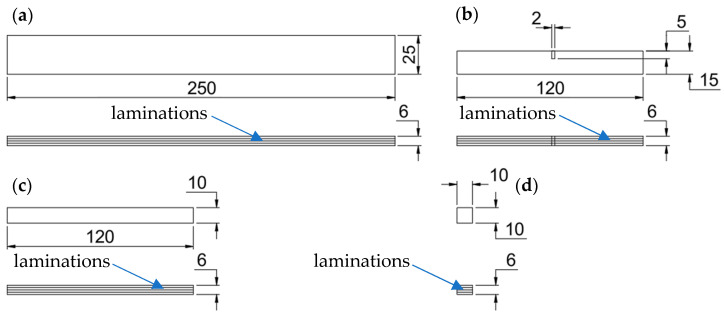
(**a**) ISO 527-4/Type 3 tensile, (**b**) ISO 179-1 notched Charpy impact, (**c**) ISO 178 three-point bending, and (**d**) ISO 604 compressive.

Ten specimens were utilized for mechanical evaluations in each experiment and temperature condition. Tensile, three-point bending, compression, and notched Charpy impact test specimens of EPGC 203 were fabricated according to the measurements indicated in ISO 527-4/Type 3 [46], ISO 178 [47], ISO 604 [48], and IEC 60893-2 [49] standards, respectively. Figure 1 illustrates a schematic detailing the size of all specimens. Tensile characterization was performed using a 100 kN universal testing machine (Instron, Norwood, MA, USA) in accordance with ISO 527-4. The tests were conducted at room temperature and 120 °C. The elastic modulus was determined at a crosshead speed of 1 mm/min using an extensometer for precise strain measurement. Subsequently, tensile strength was measured at a crosshead rate of 2 mm/min. In the tests performed at 120 °C, however, an extensometer was not used due to the elevated temperature testing conditions, and strain-related measurements were obtained from the crosshead displacement instead. Due to the use of crosshead displacement at 120 °C, these strain and modulus values are considered apparent properties incorporating system compliance. While these data effectively demonstrate the trend of stiffness degradation under thermal exposure, they should be interpreted as indicative measures rather than absolute values directly comparable to the extensometer-based measurements at room temperature. The flexural properties were determined by following ISO 178 using a three-point bending configuration with a 5 kN universal testing machine (Instron, Norwood, MA, USA). The flexural experiments consist of determining the flexural strength and modulus of the material at room temperature and at 120 °C. The flexural modulus was measured by positioning the extensometer’s tip at the center of the test specimens, with the experiments conducted at a constant rate of 1 mm/min. However, the crosshead displacement was used to determine the flexural modulus in the experiments conducted at 120 °C. Following the determination of the flexural modulus, three-point bending tests were performed at a rate of 2 mm/min. The experiments were carried out with the load applied perpendicular to the plane of the laminations. Statistical comparisons between the room-temperature and 120 °C results were performed using an independent two-sample *t*-test in Origin 2018. Statistical significance was assessed at a significance level of *p* < 0.05. Compressive strength was measured according to ISO 604, with the load applied perpendicular to the laminations. Compressive strength experiments performed at a constant rate of 1 mm/min with a 100 kN universal testing machine, (Instron, Norwood, MA, USA). Impact strength was evaluated utilizing the notched Charpy test method as specified in ISO 179-1 [50] and ISO 179-2 [51] using an Ceast 9050 impact tester (Instron, Norwood, MA, USA). Tests were performed parallel to the laminations. The test was performed using a 50 J pendulum hammer.

Fatigue behavior was evaluated under controlled cyclic loading conditions to investigate the long-term durability of the material in compliance with ISO 13003 [52]. Fatigue tests were conducted at room temperature and at 120 °C to assess the combined influence of cyclic loading and temperature on fatigue performance. Fatigue specimens were prepared according to the ISO 527-4/Type 3 standard dimensions, using the same geometry employed in the tensile tests. The tests were performed using a 250 kN servo-hydraulic testing machine (Instron, Norwood, MA, USA). An extensometer was attached to the gauge length of each specimen to measure elongation at room temperature, while traverse displacement was used to record elongation at 120 °C. The fatigue tests were carried out in stress-controlled mode. A fully reversed loading condition with a stress ratio of R=−1 was used in the experiments to simulate alternating tension–compression loading. The initial maximum stress level was selected as approximately 50% of the UTS determined from the tensile tests. Subsequently, the stress level was reduced to obtain fatigue lives in the range of 10^2^, 10^3^, 10^4^, 10^5^, and 10^6^ cycles. For each stress level, at least five specimens were tested to ensure reproducibility and statistical consistency. Fatigue loading was initiated at a nominal frequency of 0.5 Hz and then gradually increased by 0.5 Hz per second until the testing frequency of 5 Hz was reached to avoid specimen self-heating. This gradual frequency increase was adopted to minimize force-control instabilities and improve measurement accuracy. Preliminary trials were conducted to establish suitable loading conditions and to achieve the target cycle ranges. The number of cycles to failure was recorded for each test, and the resulting data were used to construct S-N curves and determine the fatigue strength of the material under the investigated conditions.

Following impact and fatigue testing, the specimens were examined using a SMZ800N stereomicroscope (Nikon, Tokyo, Japan). The acquired optical images were used for qualitative assessment of macroscopic damage features and fracture characteristics of the tested specimens, including visible cracking, edge damage, delamination, and overall fracture appearance.

## 3. Results and Discussion

### 3.1. Mechanical Properties

**Figure 2:**
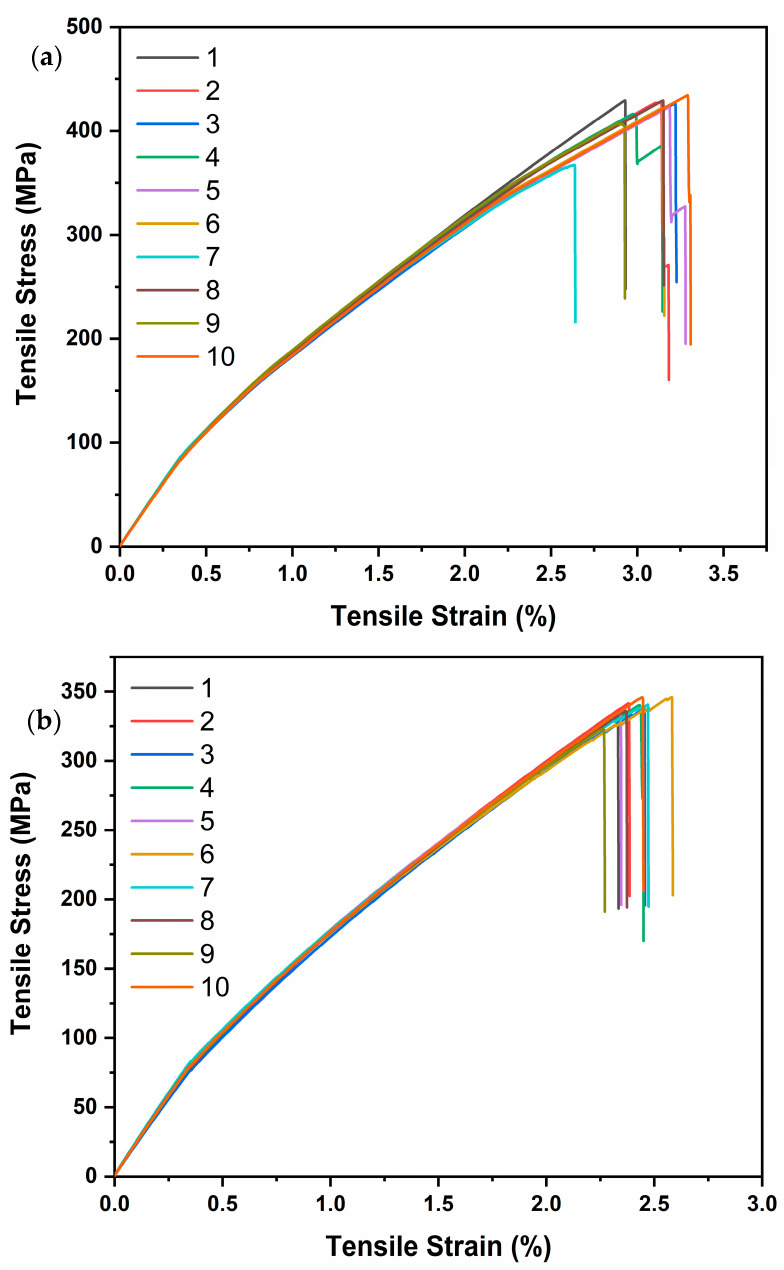
Tensile stress–strain curves of EPGC 203 at (**a**) room temperature and (**b**) 120 °C.

The tensile behavior of the EPGC 203 was evaluated at room temperature and 120 °C to assess the effect of temperature on its mechanical performance, as shown in Figure 2a,b. In both conditions, the stress–strain curves exhibited a similar overall evolution, characterized by a continuous increase in stress with increasing strain and the absence of a distinct yield limit. This behavior is consistent with strain-hardening dominated deformation mechanisms reported for fiber-reinforced composites, where progressive matrix deformation and fiber reorientation contribute to maintaining a stable load-carrying capability during increasing strain levels [53]. The close alignment of the individual curves within each temperature range indicates strong repeatability and a relatively uniform mechanical response among the tested specimens.

A temperature sensitivity was observed in both strength and deformability. At room temperature, the specimens exhibited an average ultimate tensile strength of 418.45 MPa with a standard deviation of 19.56 MPa. At 120 °C, the tensile strength decreased to an average value of 337.83 MPa with a standard deviation of 6.79 MPa, indicating a reduction in load-carrying capacity at elevated temperature. The observed reduction of nearly 20% in tensile strength at elevated temperature may be associated with temperature-dependent changes in the epoxy matrix response and possible reductions in fiber–matrix load-transfer efficiency, which reduce stress transfer efficiency and accelerate structural degradation processes within the composite [54,55,56]. The tensile modulus showed only a limited reduction with increasing temperature, decreasing from an average value of 24.48 GPa at room temperature to 23.38 GPa at 120 °C. This relatively small change suggests that the initial stiffness of the composite remained largely governed by the glass fiber reinforcement, while the elevated temperature predominantly affected the matrix-dominated deformation and failure mechanisms [57]. In parallel with the reduction in tensile strength, the tensile strain also decreased at 120 °C, falling from an average value of 3.05% at room temperature to 2.40%. Although the magnitude of this reduction is relatively moderate, it indicates a deterioration in the deformation capability of the composite under elevated-temperature conditions [58]. Furthermore, the sharper stress drop observed near final fracture at 120 °C suggests that damage accumulation and crack propagation became less stable with increasing temperature, resulting in a more abrupt failure behavior [59]. Nevertheless, the standard deviation among the tested samples remained relatively low under both conditions confirming that the observed reduction in mechanical performance was primarily caused by the temperature effect.

**Figure 3:**
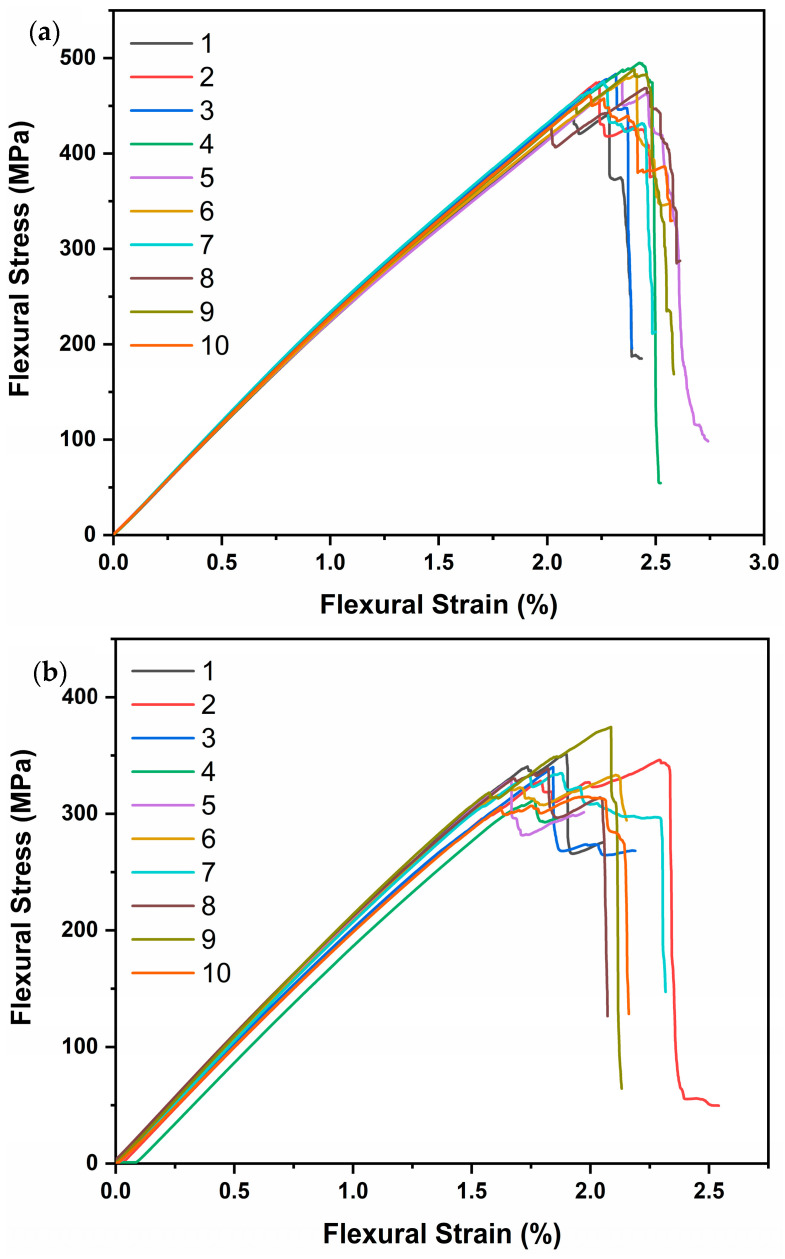
Flexural stress–strain curves of EPGC 203 at (**a**) room temperature and (**b**) 120 °C.

Figure 3a,b demonstrate the flexural strain vs. flexural stress results of EPGC 203 at room temperature and 120 °C. In both conditions, the flexural response was characterized by a progressive increase in stress with increasing strain, reflecting the complex stress distribution generated through the laminate thickness during bending. In contrast to uniaxial tensile loading, flexural deformation involves simultaneous tensile and compressive stresses together with interlaminar shear effects, which contribute to the observed nonlinear behavior prior to final failure [60].

The flexural response of EPGC 203 exhibits a pronounced temperature dependence in both stiffness and strength. At room temperature, the material shows a steep initial linear region, reaches an average flexural stress of 474.83 MPa with a standard deviation of 15.55 MPa, and then undergoes a sudden post-peak load drop, indicating high flexural rigidity and a brittle to quasi-brittle failure mode. In contrast, at 120 °C, the peak flexural stress decreased to an average value of 337.62 MPa with a standard deviation of 17.93 MPa, while the curves showed an earlier transition from linear behavior and a less uniform post-peak response. This behavior is characteristic of glass-reinforced thermosetting laminates, which generally provide high stiffness and strength under bending but exhibit limited plastic deformation prior to failure [61,62].

The reduction in flexural capacity at elevated temperature may be related to temperature-dependent changes in the matrix-dominated deformation response and possible reductions in interfacial load-transfer efficiency between the glass fibers and matrix [63]. Under flexural loading, possible contributing damage processes may include matrix cracking, interlaminar damage, and progressive fiber/matrix debonding; however, these mechanisms are discussed here as plausible interpretations consistent with the observed macroscopic response [64]. In addition, a step-like reduction in the load-carrying capacity was observed during bending, which can be attributed to progressive interlaminar damage and sequential fracture of the laminated structure. The observed scatter in peak strain and post-peak softening behavior among the specimens can be attributed to microstructural heterogeneity, including local defects, resin-rich regions, slight variations in laminate architecture, and geometric imperfections introduced during manufacturing or testing. Such specimen-to-specimen variability is commonly reported in fiber-reinforced thermosetting composites, where flexural performance is strongly influenced by void content, ply quality, and localized stress concentrations near the tensile side of the laminate [65].

**Figure 4:**
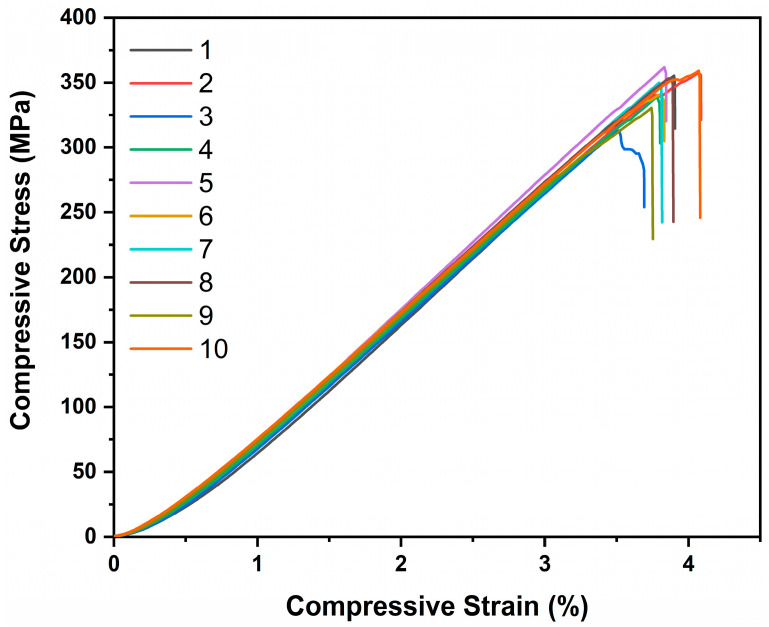
Compressive stress–strain curves of EPGC 203.

The compressive stress–strain responses of EPGC 203 are presented in Figure 4. The material exhibited stable and progressively hardening behavior throughout most of the loading range. The stress–strain curves showed strong overlap in the initial and intermediate deformation regions, indicating a relatively homogeneous material response under compressive loading. Unlike brittle materials that fail at relatively low compressive strain levels, the investigated laminate sustained substantial deformation before reaching its maximum compressive stress, demonstrating a significant load-carrying capability under compression. Such behavior is commonly observed in fiber-reinforced thermosetting composites, where progressive matrix deformation and fiber-supported stress redistribution contribute to enhanced compressive stability [66]. The compressive stress increased almost linearly with increasing strain throughout most of the loading process, reaching an average compressive strength of 342.10 MPa with a standard deviation of 22.20 MPa. Failure occurred at an average compressive strain of 3.86%, although a moderate specimen-to-specimen variation in the final fracture point was observed. After the peak stress was reached, the stress drop occurred rapidly, indicating unstable failure following the critical compressive damage state. In several specimens, slight nonlinear behavior developed near the peak region, which may indicate the onset of local damage development within the laminate structure. Possible mechanisms may include matrix cracking, interfacial damage, and localized shear deformation [67,68].

**Figure 5:**
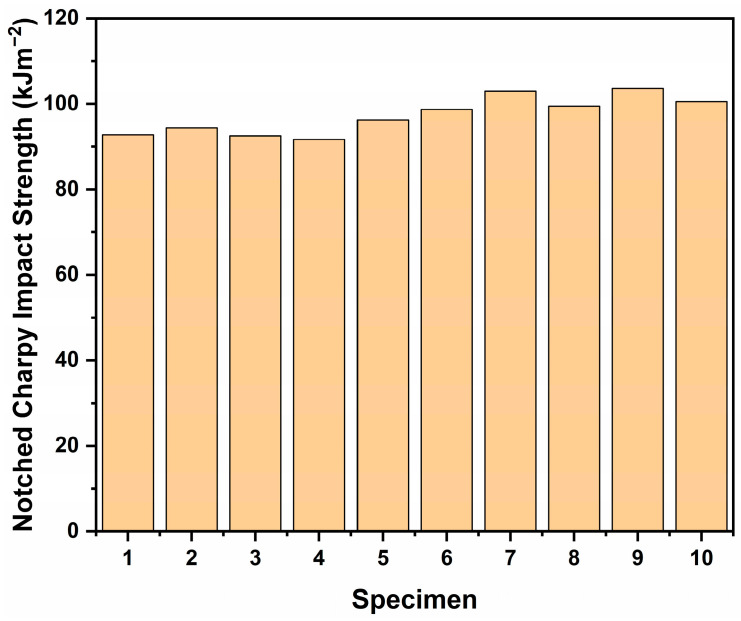
Notched Charpy impact strength of EPGC 203.

Figure 5 presents the notched Charpy impact strength results of EPGC 203. The material exhibited an average impact strength of 97.30 kJ/m^2^ with a standard deviation of 4.16 kJ/m^2^, while the absorbed impact energy showed an average value of 6.17 J with a standard deviation of 0.29 J.

**Figure 6:**
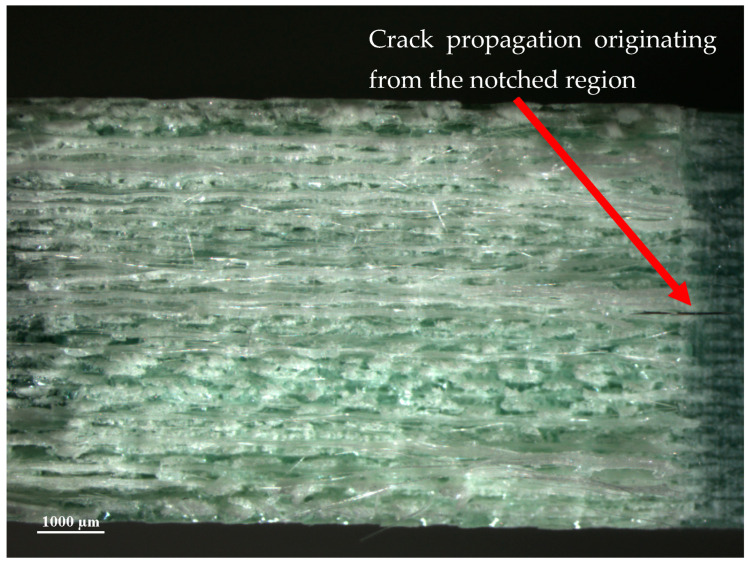
Optical image of the EPGC 203 specimen after the notched Charpy impact test.

Although minor variations were observed, these differences did not significantly influence the overall interpretation of the results. Such variations are commonly associated with slight differences in notch geometry, local microstructural heterogeneity, void distribution, and the experimental characteristic of impact testing in laminated composite materials [69,70]. Visual inspection of the tested specimen further supports the experimentally observed impact response, as shown in Figure 6. The fracture pattern is predominantly brittle, with crack propagation initiating from the notched region and limited apparent plastic deformation in the surrounding area [71]. The observed cross-sectional damage includes localized interlaminar separations and locally exposed fiber bundles, suggesting that energy dissipation occurred primarily through crack formation and propagation accompanied by internal layer-wise damage development [72]. The similarity of the damage features among the tested specimens indicates a consistent impact failure mode.

To improve the clarity and comparability of the mechanical results, the mean values, standard deviations, number of specimens, testing temperatures, retention at 120 °C, and statistical significance for the temperature-dependent properties are given in Table 2.

Statistical comparison between room temperature and 120 °C confirmed that the reductions in tensile strength, tensile modulus, flexural strength, and flexural modulus were all statistically significant (*p* < 0.001). Tensile strength and tensile modulus retained 80.73% and 95.49% of their room-temperature values at 120 °C, respectively, whereas flexural strength and flexural modulus retained 71.10% and 90.33%. Overall, the results demonstrate that elevated temperature significantly affects both strength and stiffness, with strength-related properties exhibiting a more pronounced thermal sensitivity than modulus-related parameters.

### 3.2. Fatigue Behavior

**Figure 7:**
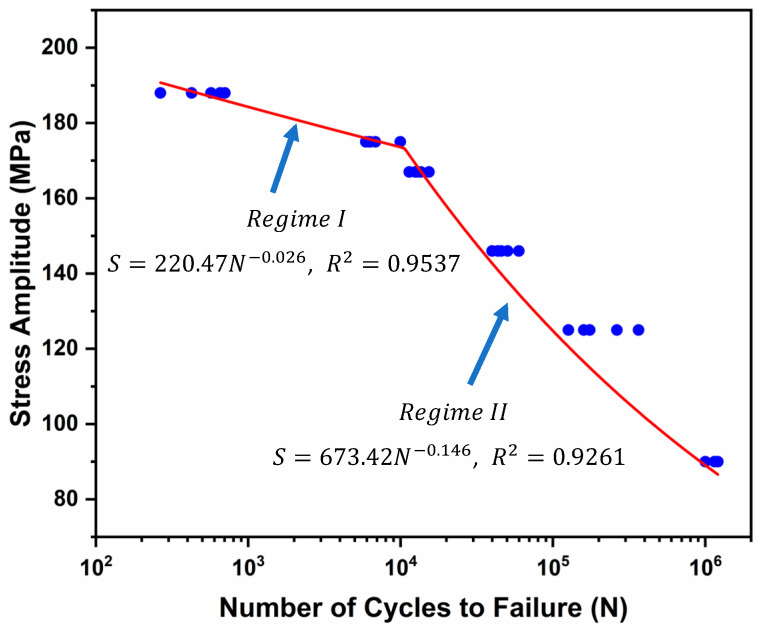
S-N fatigue behavior of EPGC 203 at room temperature with Basquin-law fitting curves.

**Figure 8:**
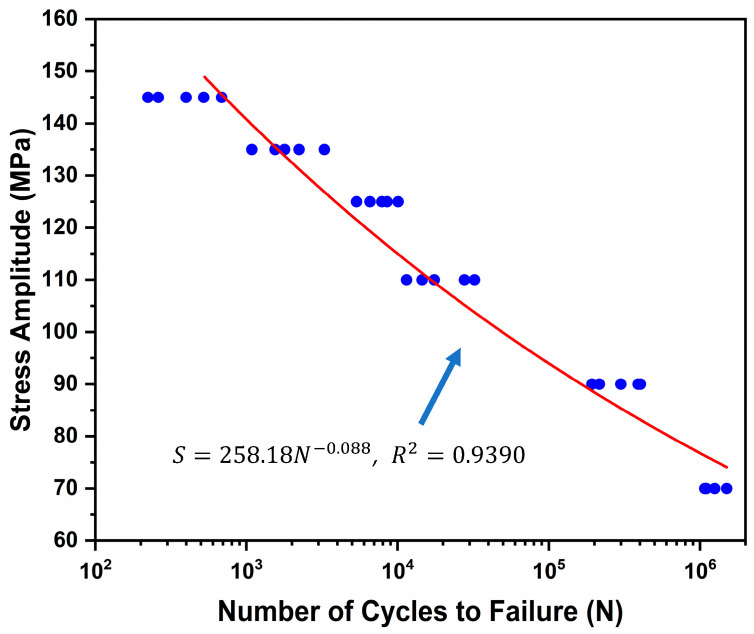
S-N fatigue behavior of EPGC 203 at 120 °C with Basquin-law fitting curve.

Figure 7 and Figure 8 show the fatigue S-N behavior of EPGC 203 under cyclic loading at room temperature and 120 °C. In both temperature conditions, the material exhibits the typical stress-dependent fatigue response commonly observed in fiber-reinforced thermosetting composites, where fatigue life increases progressively with decreasing stress amplitude [73]. The experimental data follow a consistent downward trend and were analyzed using Basquin’s law of fatigue, expressed in Equation (1) as:

(1) $$S=aN^{b}$$

where *S* is the applied stress amplitude, N is the number of cycles to failure, and a and b are fitting constants obtained from nonlinear regression analysis. The fitted curves indicate that the fatigue degradation behavior of the material can be reasonably described by Basquin’s law.

At room temperature, the specimens subjected to the amplitude of 188 MPa failed at 10^2^ cycles whereas lower amplitudes progressively extended the fatigue life, reaching 10^5^ cycles at 125 MPa and reached 10^6^ cycles at 90 MPa. This trend indicates a classic fatigue response, in which damage accumulation accelerates substantially at higher stress levels but becomes much slower as the load amplitude is reduced. The fatigue response at room temperature exhibited a partially bilinear tendency, suggesting the presence of distinct fatigue degradation regimes during cyclic loading. Consequently, piecewise Basquin-type fitting was applied for the room temperature condition to represent the observed transition in fatigue behavior more accurately. This behavior may indicate a transition between different fatigue damage regimes during cyclic loading. A possible interpretation is a shift from early matrix-dominated damage development toward more progressive interlaminar damage accumulation at higher cycle regions [74].

A comparable fatigue behavior was observed at 120 °C; however, the material exhibited reduced fatigue resistance throughout the investigated life range. Under the highest tested stress amplitude of 145 MPa, failure occurred after approximately 10^2^ cycles, whereas stress amplitudes of 135 MPa and 125 MPa resulted in intermediate fatigue lives within the 10^3^–10^4^ cycle range. At 110 MPa, the fatigue life increased further into 10^4^ cycles, while specimens tested at 90 MPa reached fatigue lives in the order of 10^5^ cycles. At the lowest applied stress amplitude of 70 MPa, the specimens reached 10^6^ cycles. The 120 °C condition showed a more continuously decreasing stress–life relationship without a pronounced transition region. This difference suggests that elevated temperature modifies the fatigue damage evolution process and leads to a more uniform stress–life relationship throughout cyclic loading [75]. The reduced fatigue resistance at elevated temperature is also consistent with the tensile and flexural results discussed previously, indicating that temperature-dependent changes in matrix-dominated response and load-transfer behavior likely contribute to the degradation of the overall mechanical performance. It should be noted that the elevated-temperature tests were conducted at 120 °C, which remains below the reported glass transition temperature of the laminate (T_g_ = 160 °C) and its Thermal Class F rating (155 °C). Therefore, the observed reductions in mechanical and fatigue performance are considered consistent with temperature-dependent changes occurring below the glass transition region, rather than with operation above T_g_.

The fitted Basquin parameters and associated regression quality metrics are summarized in Table 3. A quantitative comparison of the fitting quality further justifies the use of a piecewise Basquin model at room temperature. A single-slope regression over the full fatigue range resulted in a relatively poor fit ($R^{2}=0.776$), indicating that the S-N response could not be satisfactorily represented by a single power-law relationship. In contrast, the piecewise Basquin approach significantly improved the correlation, yielding $R^{2}=0.9537$ for Regime I and $R^{2}=0.9261$ for Regime II. These results quantitatively support the use of a segmented fit at room temperature. At 120 °C, however, the fatigue data were well described by a single Basquin fit ($R^{2}=0.9390$), suggesting a more uniform stress–life relationship over the investigated loading range.

**Figure 9:**
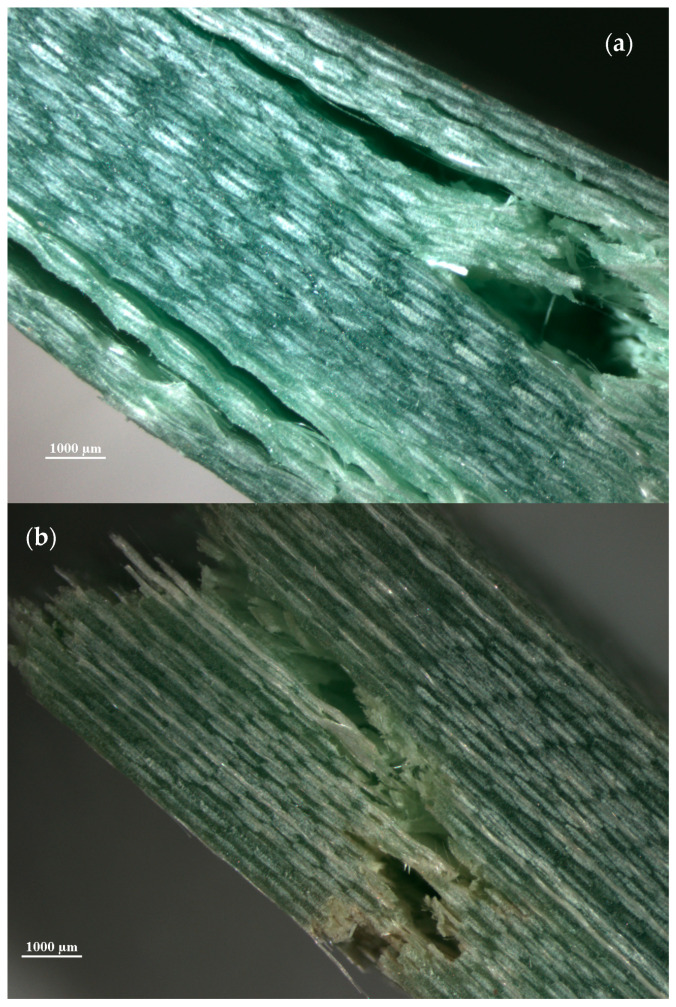
Optical post-fracture images of fatigue specimens at 10^5^ cycles: (**a**) room temperature and (**b**) 120 °C.

Representative optical images of the fatigue-tested specimens, presented in Figure 9a,b for the room temperature and 120 °C, revealed clear internal damage features. Under room temperature conditions, the fracture region exhibited distributed layer-wise cracking, interlaminar separations, and localized fiber-bundle exposure, indicating progressive damage accumulation during cyclic loading. At 120 °C, similar internal damage features were observed, together with more pronounced crack-like openings along the fracture path. These observations support the interpretation that fatigue failure developed through progressive crack initiation and propagation accompanied by internal layer-wise damage evolution at the macroscopic scale accessible by optical microscopy.

**Figure 10:**
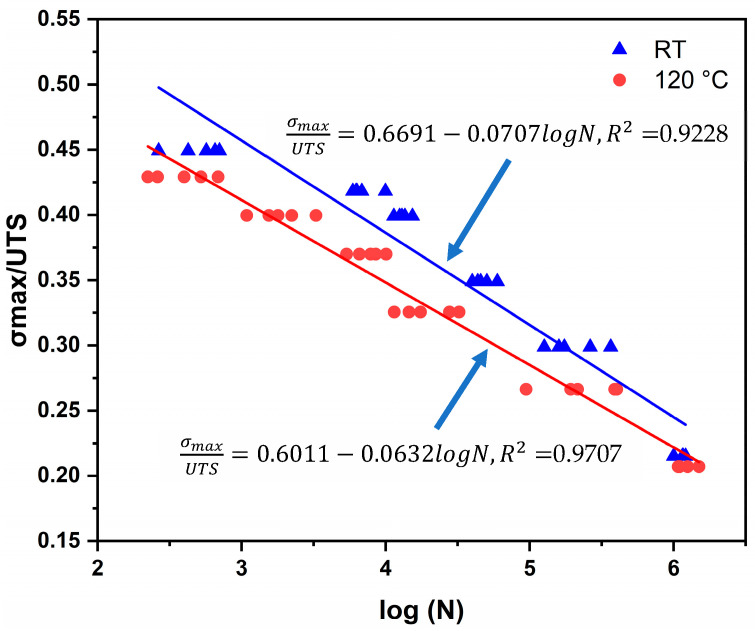
Normalized fatigue behavior of EPGC 203 at room temperature and 120 °C based on the Lorenzo–Hahn approach.

Figure 10 demonstrates the normalized fatigue behavior of EPGC 203 at room temperature and 120 °C based on the stress normalization approach proposed by Lorenzo and Hahn, where the applied cyclic stress was normalized with respect to the corresponding ultimate tensile strength of the material. The normalized fatigue response was evaluated according to the relationship given in Equation (2):

(2) $$\frac{\sigma_{max}}{UTS}=A-clog\left(N\right)$$

where $\sigma_{max}$ is the maximum applied cyclic stress, *UTS* is the ultimate tensile strength obtained from the static tensile tests, N is the number of cycles to failure, and c is the fatigue degradation coefficient associated with the slope of the normalized S-N relationship. Since the fatigue tests were conducted under a stress ratio of R=−1, the stress amplitude $\sigma_{a}$ is equal to the maximum applied stress.

The normalized fatigue curves obtained at room temperature and 120 °C remain relatively close to each other throughout the investigated life range, suggesting that a substantial portion of the temperature-induced fatigue degradation is directly associated with the reduction in static tensile strength at elevated temperature. This observation is consistent with the tensile and flexural results discussed previously, suggesting that elevated temperature affects matrix-dominated deformation and load-transfer behavior [76]. Nevertheless, the 120 °C data consistently exhibit slightly lower normalized fatigue levels and a more uniform linear degradation trend compared to room temperature. This behavior indicates that elevated temperature not only reduces the static load-carrying capability of the laminate but also accelerates cyclic damage accumulation mechanisms during fatigue loading.

The Lorenzo–Hahn fitting parameters are presented in Table 4. At room temperature, the normalized fatigue relationship yielded an intercept of 0.6691 and a slope (c) of −0.0707, with $R^{2}=0.9228$. At 120 °C, the corresponding values were 0.6011 and −0.0632, with a fit quality of $R^{2}=0.9707$. The high coefficients of determination confirm that the Lorenzo–Hahn normalization provides a robust description of the normalized fatigue response under both temperature conditions. The lower intercept at 120 °C indicates a reduction in normalized fatigue strength, while the slightly less negative slope suggests a modest change in the fatigue degradation trend at elevated temperature.

To provide a clearer representation of the experimental fatigue dataset, Table 5 summarizes the statistical scatter of fatigue life at each applied stress amplitude for room temperature and 120 °C. In both conditions, the number of cycles to failure increases as the stress amplitude decreases. The results also show greater scatter at lower stress amplitudes, particularly in the long-life regime, which is characteristic of the fatigue response of composite materials. In addition, the fatigue lives at 120 °C are generally lower than those measured at room temperature at comparable stress levels, indicating reduced fatigue resistance at elevated temperature.

## 4. Conclusions

This study experimentally investigated the temperature-dependent mechanical and fatigue behavior of IEC 60893 EPGC 203 woven glass fiber-reinforced epoxy laminate through tensile, flexural, compressive, impact, and fatigue testing performed at room temperature and 120 °C.

- Increasing the temperature from room temperature to 120 °C reduced the tensile and flexural strengths of the material, which is consistent with temperature-dependent changes in matrix-dominated response and load-transfer behavior.
- Flexural and compressive tests revealed predominantly brittle to quasi-brittle failure behavior characterized by abrupt post-peak stress drops and progressive damage development.
- Charpy impact tests demonstrated stable notched impact resistance and consistent fracture behavior, indicating reliable resistance against sudden and transient mechanical loading conditions.
- Fatigue results showed that the material retained cyclic endurance capability under a stress ratio of *R* = −1, achieving fatigue lives up to 10^6^ cycles at lower stress amplitudes. Elevated temperature accelerated fatigue degradation and reduced fatigue strength throughout the investigated life range, indicating increased sensitivity to cyclic damage accumulation under combined thermal and mechanical loading.
- Basquin and Lorenzo–Hahn analyses demonstrated that the fatigue response of EPGC 203 can be effectively represented using stress–life modeling approaches, while the normalized fatigue analysis confirmed that thermal exposure influences both static strength and cyclic durability.

## Figures and Tables

**Table 1:** Material specifications of the investigated EPGC 203.

Parameters	Description
Fiber orientation	0°/90°
Glass fiber content	~70 wt.%
Density	1.85 g/cm^3^
T_g_	160 °C
Thermal class	Class F (155 °C)
Insulation resistance	10^14^ Ω
Specific surface resistance	5 × 10^10^ Ω
Comparative tracking index	180 CTI

**Table 2:** Summary of the mechanical properties of EPGC 203.

Property	Temperature	n	Mean ± SD	Retention at 120 °C	Statistical Significance
Tensile Strength	RT	10	418.45 ± 19.56 MPa	Reference	-
Tensile Strength	120 °C	10	337.83 ± 6.79 MPa	80.73%	*p* < 0.001
Tensile Modulus	RT	10	24,478 ± 248.27 MPa	Reference	-
Tensile Modulus *	120 °C	10	23,375 ± 563.19 MPa	95.49%	*p* < 0.001
Flexural Strength	RT	10	474.83 ± 15.55 MPa	Reference	-
Flexural Strength	120 °C	10	337.62 ± 17.93 MPa	71.1%	*p* < 0.001
Flexural Modulus	RT	10	23,417 ± 284.19 MPa	Reference	-
Flexural Modulus *	120 °C	10	21,152 ± 327.51 MPa	90.33%	*p* < 0.001
Compressive Strength	RT	10	342.1 ± 22.20 MPa	N/A	N/A
Impact Strength	RT	10	97.30 ± 4.16 kJ/m^2^	N/A	N/A

* The tensile and flexural modulus values reported at 120 °C are apparent values.

**Table 3:** Basquin fitting parameters and regression quality metric for the fatigue data.

Temperature	Fit Type	Regime	*a*	*b*	*R* ^2^
RT	Single	Full range	417.29 ± 34.154	−0.106 ± 0.00883	0.7760
RT	Piecewise	Regime I	220.47 ± 3.356	−0.026 ± 0.00202	0.9537
RT	Piecewise	Regime II	673.42 ± 60.567	−0.146 ± 0.00874	0.9261
120 °C	Single	Full range	258.18 ± 9.644	−0.088 ± 0.00419	0.9390

**Table 4:** Lorenzo–Hahn fitting parameters and regression quality metric for normalized fatigue behavior.

Temperature	Intercept (*A*)	Slope (*c*)	*R* ^2^
RT	0.6691 ± 0.01767	−0.0707 ± 0.00386	0.9228
120 °C	0.6011 ± 0.00914	−0.0632 ± 0.00207	0.9707

**Table 5:** Statistical scatter of fatigue life at each stress level.

Temperature	Stress Amplitude	n	Mean *N*	SD	Range of *N*
RT	188 MPa	5	522.8	171.3	265–702
RT	175 MPa	5	7046.4	1662.9	5909–9962
RT	167 MPa	5	13,157.4	1461.5	11,398–15,345
RT	146 MPa	5	47,857.0	7641.1	39,874–59,691
RT	125 MPa	5	217,641.2	96,697.6	126,262–365,082
RT	90 MPa	5	1,104,364.4	97,592.4	1,000,000–1,209,603
120 °C	145 MPa	5	418.2	190.8	223–686
120 °C	135 MPa	5	1986.6	833.9	1086–3282
120 °C	125 MPa	5	7679.6	1814.5	5356–10,085
120 °C	110 MPa	5	20,714.2	8912.4	11,489–32,328
120 °C	90 MPa	5	299,855.6	96,377.9	192,785–402,621
120 °C	70 MPa	5	1,202,574.8	180,749.0	1,075,489–1,500,035

## Data Availability

The article includes the necessary data; further inquiries can be directed to the corresponding author.

## Abbreviations

The following abbreviations are used in this manuscript:

CNC	Computer numerical control
EPGC	Epoxy woven glass cloth
HV	High voltage
HVDC	High-voltage direct current
n	Number of tested specimens
*N*	Number of cycles to failure
RT	Room temperature
SD	Standard deviation
S-N	Stress–life
UTS	Ultimate tensile strength
*σ_a_*	Stress amplitude
*σ_max_*	Maximum applied cyclic stress
